# Proximal vs. total gastrectomy for proximal advanced gastric cancer after neoadjuvant chemotherapy: a systematic review and meta-analysis of propensity score-matched studies

**DOI:** 10.3389/fonc.2026.1805000

**Published:** 2026-06-02

**Authors:** Yueping Li, Xiaofei Nie, Lianda Zhang, Yan Liang, Chengcong Liu

**Affiliations:** 1 Department of General Surgery, Qingdao Central Hospital, University of Health and Rehabilitation Sciences, Qingdao, China; 2 Department of Oncology & Radiotherapy, Qingdao Central Hospital, University of Health and Rehabilitation Sciences, Qingdao, China; 3 Department of General Internal Medicine, Qingdao Central Hospital, University of Health and Rehabilitation Sciences, Qingdao, China

**Keywords:** Meta-analysis, neoadjuvant chemotherapy, propensity score matching, proximal gastrectomy, proximal gastric cancer, total gastrectomy

## Abstract

**Background:**

The optimal extent of gastrectomy for proximal advanced gastric cancer (PAGC) after neoadjuvant chemotherapy (NAC) remains controversial. While total gastrectomy (TG) has traditionally been considered oncological safer, proximal gastrectomy (PG) offers potential functional advantages. This meta-analysis aimed to compare short- and long-term outcomes between PG and TG after NAC.

**Methods:**

We systematically searched PubMed, Embase, Web of Science, and the Cochrane Library for studies comparing PG and TG in PAGC patients treated with NAC. Primary outcomes included overall survival (OS), recurrence-free survival (RFS), and postoperative complications. Supplementary TG-cohort data were extracted to assess distal lymph node metastasis patterns. Hazard ratios (HRs), odds ratios (ORs), and mean differences (MDs) with 95% confidence intervals (CIs) were calculated using fixed- or random-effects models as appropriate. Publication bias and result robustness were assessed through funnel plots, Galbraith plots, L’Abbé plots, and sensitivity analyses.

**Results:**

Four propensity score-matched (PSM) comparative studies were included in the quantitative meta-analysis, comprising 284 patients undergoing PG and 305 patients undergoing TG after NAC. Baseline characteristics were comparable between groups. PG was associated with shorter operative time (MD −21.4 min, 95% CI −23.7 to −19.1) but slightly greater blood loss (MD 8.9 mL, 95% CI 4.9–12.8). Fewer lymph nodes were retrieved with PG (MD −3.9, 95% CI −4.5 to −3.3), but the number of metastatic nodes was similar (MD −0.27, 95% CI −0.63 to 0.09). Supplementary TG-cohort analysis showed low metastasis rates in distal lymph node stations (#4: 12.7%, #5: 4.8%, #6: 5.1%, and #12: 2.2%). Overall postoperative complications did not differ between PG and TG (OR 1.05, 95% CI 0.71–1.54), nor did Clavien-Dindo grade ≥ II complications (OR 1.08, 95% CI 0.69–1.71). No significant differences were observed in 5-year OS (HR 1.23, 95% CI 0.83–1.84) or RFS (HR 1.36, 95% CI 0.94–1.95). Sensitivity analyses supported the stability of the main pooled estimates.

**Conclusions:**

In patients with PAGC following NAC, PG provides comparable long-term oncological outcomes to TG without increasing postoperative morbidity or compromising adjuvant treatment delivery. PG represents a feasible oncological alternative to TG in appropriately selected patients.

**Systematic Review Registration:**

https://www.crd.york.ac.uk/prospero/, identifier CRD420261388093.

## Introduction

Gastric cancer (GC) remains a major global health burden and continues to rank among the most common and lethal malignancies worldwide (1). According to recent GLOBOCAN estimates, approximately 969,000 new GC cases and 660,000 GC-related deaths occurred in 2022, making GC the fifth most frequently diagnosed cancer and one of the leading causes of cancer mortality globally (1–3).  Despite advances in screening, perioperative management, and systemic therapy, the disease burden remains substantial, with marked geographic variation and a particularly high incidence in East Asian populations (1, 4, 5). Within this heterogeneous disease spectrum, proximal GC has shown a relative increase in incidence and poses distinct surgical challenges, as treatment must balance oncological radicality with preservation of postoperative function and quality of life (1, 6–8).

For patients with resectable locally advanced disease, multimodal treatment integrating neoadjuvant chemotherapy (NAC) followed by surgery has become an established therapeutic strategy (9–11). NAC aims to downstage the primary tumor, reduce nodal tumor burden, eradicate micrometastatic disease, and increase the likelihood of achieving R0 resection, thereby improving long-term outcomes (12–14). In patients who respond favorably to NAC, surgical resection remains the cornerstone of curative-intent treatment. However, the optimal extent of gastrectomy for proximal advanced GC (PAGC) after NAC—particularly the choice between proximal gastrectomy (PG) and total gastrectomy (TG)—remains controversial and has not reached international consensus (15, 16).

PG preserves the distal stomach and may offer physiological and functional advantages, including reduced risks of postgastrectomy nutritional deficiency, anemia, and dumping syndrome compared with TG (7, 8, 17). These potential benefits make PG an attractive option for surgeons seeking to improve postoperative recovery and long-term quality of life. In contrast, TG, which involves complete gastric removal with D2 lymphadenectomy, has traditionally been regarded as a more radical and oncologically secure procedure. By removing the entire stomach, TG is thought to reduce the risk of remnant gastric recurrence and permit more comprehensive lymph node clearance. The central clinical dilemma is therefore whether the functional benefits of PG can be achieved without compromising oncological safety, particularly in patients whose tumors have been downstaged by NAC but may still retain aggressive biological features.

Existing comparative studies and meta-analyses of PG versus TG have yielded inconsistent conclusions, largely because of heterogeneous patient populations, inclusion of early-stage disease, and, importantly, limited focus on patients treated with modern NAC regimens (18–21). NAC-induced tumor regression and anatomical changes may alter surgical planes, nodal tumor burden, and lymphatic drainage patterns, potentially influencing the feasibility, safety, and oncological adequacy of stomach-preserving surgery (10, 11). Consequently, evidence directly informing surgical decision-making in this increasingly relevant setting—PAGC after NAC—remains fragmented and insufficient.

This systematic review and meta-analysis therefore aimed to synthesize the available comparative evidence on PG versus TG after NAC in patients with PAGC, with specific attention to perioperative outcomes, long-term survival, and the oncological relevance of distal lymph node station dissection. To our knowledge, this is the first meta-analysis focused specifically on PAGC treated with NAC. By providing a targeted evidence synthesis, this study seeks to inform surgical decision-making and support more individualized, evidence-based management strategies for this challenging patient population.

## Materials and methods

This systematic review and meta-analysis was conducted and reported following the Preferred Reporting Items for Systematic Reviews and Meta-analysis (PRISMA) guidelines (22). The protocol was retrospectively registered in PROSPERO (Registration No. CRD420261388093).

### Search strategy

A comprehensive and systematic literature search was performed across four major electronic databases: PubMed, Embase, the Cochrane Library, and Web of Science. The search aimed to identify all relevant studies published from database inception until December 2025. The search strategy used a combination of Medical Subject Headings (MeSH) terms and free-text keywords related to three core concepts: (1) gastric cancer (“stomach neoplasms”, “gastric cancer”), (2) surgical procedures (“proximal gastrectomy”, “total gastrectomy”), and (3) neoadjuvant therapy (“neoadjuvant chemotherapy”, “preoperative chemotherapy”). The search syntax was adapted for each database, and the full search strategy is provided in **Supplementary Table 1**. No language restrictions were initially applied. In addition, the reference lists of all included studies and relevant reviews were manually screened to identify potentially eligible publications not captured by the electronic search. References were managed using EndNote software (version X9; Clarivate), and duplicates were manually removed.

### Study selection

Two investigators independently screened the titles and abstracts of all retrieved records according to the predefined eligibility criteria. Full-text articles of potentially relevant studies were then assessed independently, and disagreements were resolved by discussion or consultation with a third senior investigator. For the main comparative meta-analysis, studies were eligible if they met the following PICOS criteria (23): (1) Population: adult patients with histologically confirmed locally advanced proximal gastric adenocarcinoma, including Siewert type II/III or upper-third gastric cancer, who received neoadjuvant chemotherapy; (2) Intervention and Comparison: direct comparison of surgical outcomes between PG and TG; (3) Outcomes: reporting at least one primary outcome of interest, including overall survival or recurrence-free survival, or key secondary outcomes, including postoperative morbidity, mortality, operative details, or lymph node-related pathological findings; and (4) Study Design: published comparative clinical studies. Although randomized controlled trials (RCTs) and observational comparative studies were initially considered, no eligible RCTs directly comparing PG and TG after NAC was identified. Therefore, to improve comparability and reduce selection bias, the quantitative meta-analysis was restricted to propensity score-matched (PSM) studies. Reviews, case reports, conference abstracts, animal studies, non-PSM comparative studies, and studies without extractable data were excluded from the comparative meta-analysis. In addition, one TG-only single-arm study reporting distal lymph node metastasis was included solely for supplementary contextual analysis and was not incorporated into any comparative effect estimates between PG and TG.

### Data extraction

A standardized, pilot-tested data extraction form was used. Two authors independently extracted data from each included study. Extracted information included: (1) study characteristics, including first author, publication year, country, study design, and sample size; (2) patient demographics and clinical baseline data, including age, sex, body mass index (BMI), American Society of Anesthesiologists (ASA) Physical Status classification, Charlson Comorbidity Index (CCI), TNM stage, and type and cycles of neoadjuvant chemotherapy; (3) surgical details, including extent of lymphadenectomy and reconstruction method; and (4) outcome data for PG and TG groups, including dichotomous outcomes, such as postoperative morbidity, mortality, and anastomotic leakage; continuous outcomes, such as number of harvested lymph nodes, operative time, and blood loss; and time-to-event outcomes, including overall survival (OS) and recurrence-free survival (RFS). For the supplementary distal lymph node metastasis analysis, TG-cohort data were extracted separately. Distal lymph node metastasis was defined as metastasis at nodal stations routinely dissected during TG but generally omitted or incompletely dissected during PG, primarily stations #4d, #5, #6, and #12a. A third independent reviewer reassessed all extracted data, and disagreements were resolved by consensus.

### Data synthesis

The primary comparative analyses, including overall survival, recurrence-free survival, postoperative morbidity, perioperative outcomes, pathological outcomes, and adjuvant chemotherapy administration, were specified before data extraction and were restricted to propensity score-matched comparative studies. The pooled single-arm analysis of distal lymph node metastasis in TG cohorts, including the additional single-arm TG study, was performed as a supplementary exploratory analysis to provide contextual evidence regarding distal lymph node involvement.

### Quality assessment

The methodological quality and risk of bias of the included non-randomized comparative studies were appraised independently by two reviewers using the Risk Of Bias In Non-randomized Studies - of Interventions (ROBINS-I) tool (24). This tool evaluates bias across seven domains: confounding, selection of participants, classification of interventions, deviations from intended interventions, missing data, measurement of outcomes, and selection of the reported result. Each domain was judged as having “low,” “moderate,” “serious,” or “critical” risk of bias, leading to an overall risk-of-bias judgement for each study. Any disagreement was resolved by consensus.

### Statistical analysis

All statistical analyses were performed using Review Manager (RevMan) version 5.4 and R (v4.3.2)/RStudio (v4.2.2). A two-sided p-value < 0.05 was considered statistically significant. For the comparative meta-analysis of PG versus TG, dichotomous outcomes were pooled as odds ratios (ORs) with corresponding 95% confidence intervals (CIs) using the Mantel-Haenszel method. Continuous outcomes were pooled as mean differences (MDs) or standardized mean differences (SMDs) with 95% CIs using the inverse variance method. For time-to-event outcomes, hazard ratios (HRs) with 95% CIs were pooled. Heterogeneity among studies was assessed using Cochran’s Q test, with p < 0.10 indicating statistical heterogeneity, and quantified using the I² statistic. An I² value > 50% was considered indicative of substantial heterogeneity. A random-effects model was applied when substantial heterogeneity was present; otherwise, a fixed-effects model was used. For the supplementary distal lymph node metastasis analysis, TG-cohort data were analyzed separately from the comparative PG versus TG meta-analysis. Pooled proportions with 95% CIs were calculated to summarize metastasis rates at distal lymph node stations. Sensitivity analyses were performed by sequentially excluding individual studies to evaluate the robustness of the pooled results.

## Results

### Study selection and characteristics

**Figure 1:**
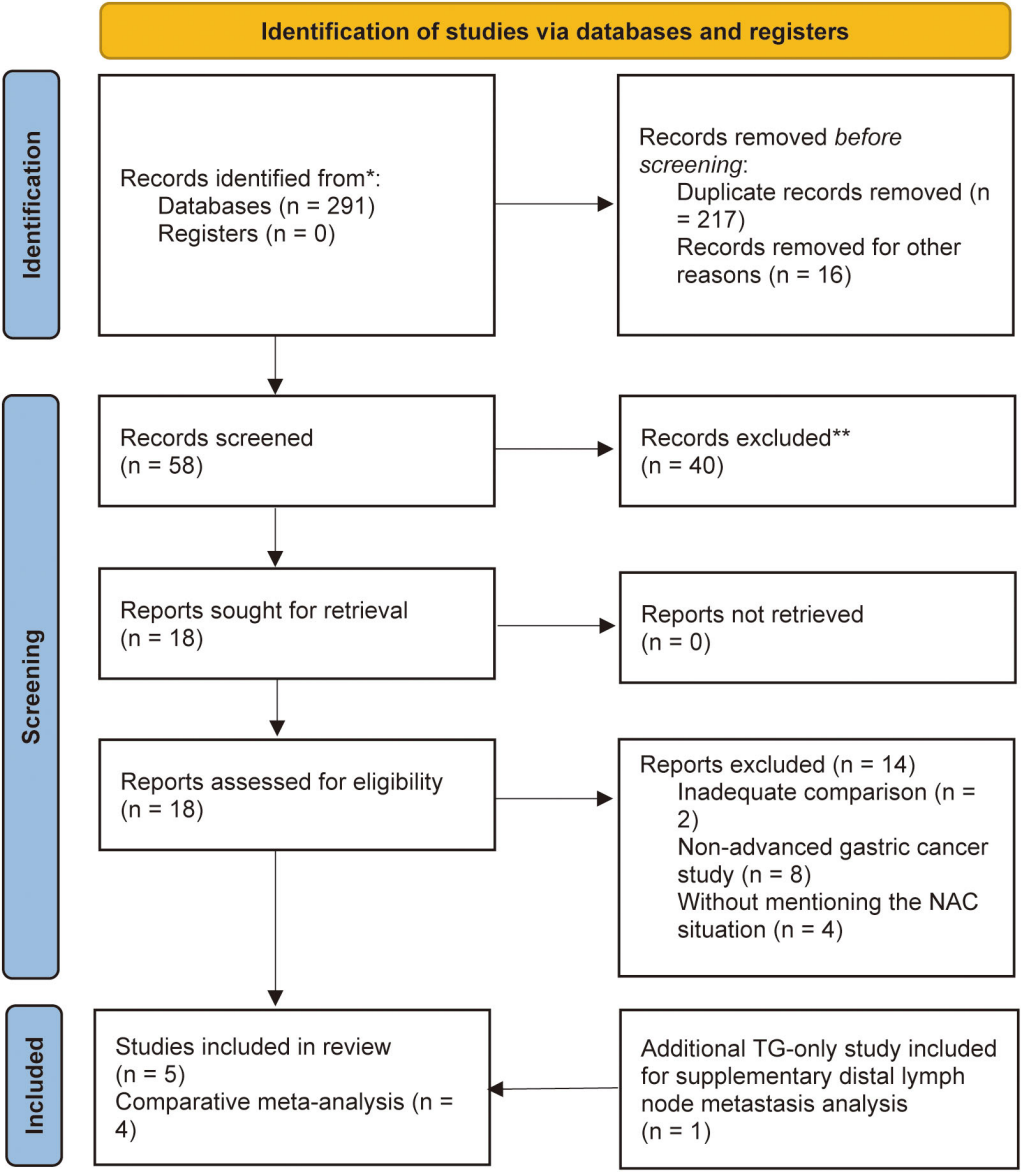
Flow chart of the search for eligible studies.

The study selection process is summarized in **Figure 1**. A systematic search across four electronic databases initially identified 291 potentially relevant records. After the removal of duplicates and screening of titles and abstracts, 18 studies were deemed eligible for full-text review based on the predefined inclusion and exclusion criteria. Following detailed assessment, four PSM comparative studies (15, 25–27) were included in the quantitative meta-analysis comparing PG and TG after NAC (**Table 1**). These studies included 284 patients in the PG group and 305 patients in the TG group. In addition, one TG-only study (28) was included separately for the supplementary exploratory analysis of distal lymph node metastasis patterns. This supplementary analysis was based on TG-cohort data from the additional TG-only study (28) and the TG arm of one included PSM comparative study (26). The additional TG-only study was not included in the comparative meta-analysis and did not contribute to any pooled effect estimates between PG and TG. Inter-reviewer agreement for study selection was excellent (κ = 0.93), and any discrepancies were resolved through discussion with a third investigator.

**Table 1:** Characteristics of included studies.

First author	Year	Study period	Study design	No. patients		Inclusion criteria
				Before PSM	After PSM	
Yuan et al.	2024	2009-2022	Multi-Center Retrospective PSM Cohort Study	274	160 PG: 80 TG: 80	(1) patients diagnosed with locally advanced GC with tumors located in the upper third of the stomach without esophagogastric junction adenocarcinomas; (2) tumor stage ranging from TNM stage I to III, with no evidence of distant metastases; (3) patients who underwent at least NACT followed by minimally invasive radical gastrectomy (laparoscopic or robotic surgery); (4) American Society of Anesthesiology (ASA) score of class I, II, or III.
Lu et al.	2025	2012-2022	Multi-Center Retrospective PSM Cohort Study	403	244 PG: 122 TG: 122	(1) Histologic confirmation of GC; (2) Tumor location in the upper third of the stomach without esophagus invasion; (3) Computed tomography indicating locally advanced GC without evidence of distant metastasis; (4) American Society of Anesthesiology (ASA) score of class I-III; (5) NACT prior to surgery; and (6) Gastric tubular reconstruction.
Gu et al.	2024	2009-2022	Single-Center Retrospective PSM Cohort Study	330	110 PG: 39 TG: 71	Patients underwent resection with curative intent after neoadjuvant therapy for histologically confirmed GC. The exact anatomic location of the proximal includes tumors arising from the upper third of the stomach, and most cancers arise 2–5 cm below the esophagogastric junction, the so-called Siewert III tumor.
Sugoor et al.	2016	2010-2012	Single-Center Retrospective PSM Cohort Study	343	75 PG: 43 TG: 32	Patients with advanced gastric cancer at the upper-third level of the stomach or EGJ cancer underwent curative PG or TG with standard LN dissection.
Chen et al.	2024	–	Multi-Center Single-arm Retrospective Cohort Study	150 TG:150	–	(i) Age between 18 and 80 years, of any sex; (ii) Histological diagnosis of gastric/esophagogastric junction adenocarcinoma in the upper part of the stomach; (iii) Received neoadjuvant chemotherapy; (iv) Received total gastrectomy with standardized D2 lymphadenectomy.

GC, gastric cancer; PG, proximal gastrectomy; PSM, propensity score-matched; TG, total gastrectomy.

### Quality assessment

**Figure 2:**
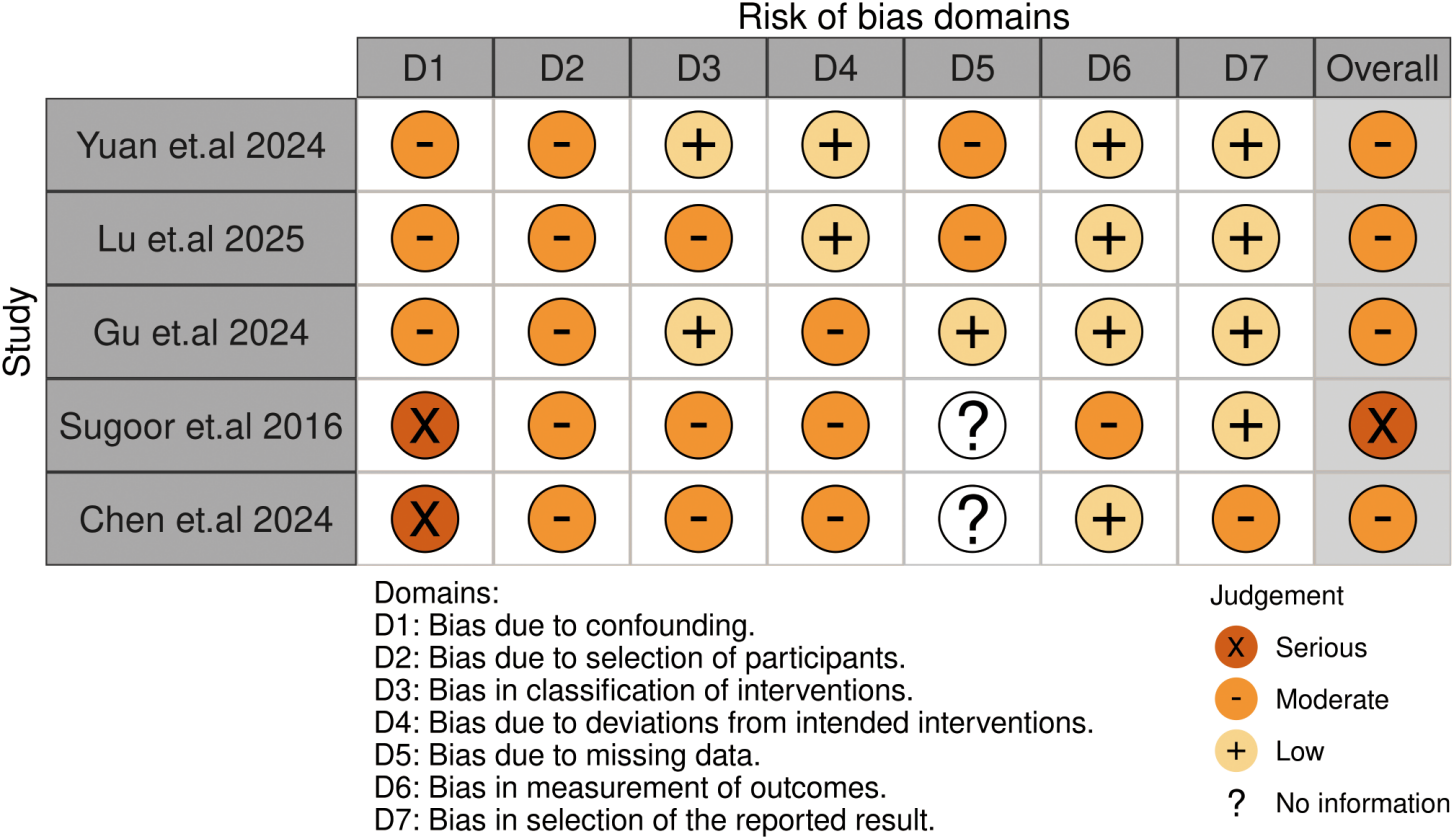
Risk of bias plot following the ROBINS- I tool for quality assessment.

The methodological quality of the four included non-randomized comparative studies was assessed using the ROBINS-I tool. Overall, three studies were judged to have a moderate risk of bias (25, 26, 29, 30), whereas one study was considered to have a serious risk of bias (31). The main methodological concerns were related to the inherent limitations of retrospective observational studies, particularly the potential for residual confounding due to unmeasured or insufficiently adjusted factors. In addition, differences in surgical expertise, perioperative management protocols, and criteria for selecting PG versus TG were not consistently reported or adjusted for across studies, which may have influenced comparative outcomes. The detailed domain-specific judgments are presented in **Figure 2**.

### Study characteristics

**Figure 3:**
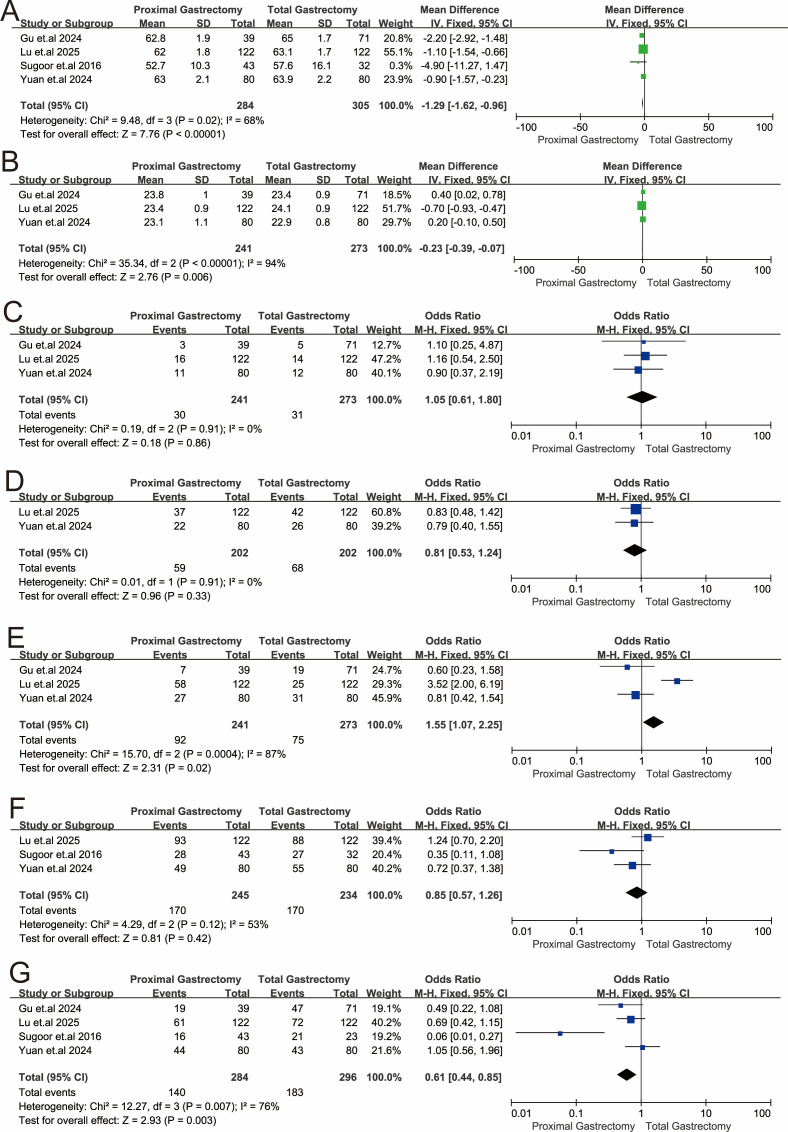
Forest plot of baseline characteristics between PG and TG groups. **(A)** Age distribution. **(B)** Body mass index. **(C)** American Society of Anesthesiologists classification (≥ 3). **(D)** Charlson Comorbidity Index (≥ 2). **(E)** Neoadjuvant chemotherapy regimen. **(F)** TNM stage. **(G)** Proportion of poorly differentiated tumors.

Pooled analysis of baseline characteristics showed that most demographic and clinical variables were generally comparable between the PG and TG groups, including age, ASA classification, CCI score, neoadjuvant regimen, and TNM stage (all P > 0.05) (**Figures 3A, C–F****;** **Table 2**). However, the PG group had a significantly lower BMI (P < 0.001; I² = 94%) and a lower proportion of poorly differentiated tumors (P = 0.007; I² = 76%) than the TG group (**Figures 3B, G**). These imbalances were considered when interpreting the pooled findings.

**Table 2:** Baseline demographics of the included studies.

Study	Age, years	Gender (Male)	BMI	ASA score (1-2/3)	CCI score (≤2/>2)	ypTNM stage				TRG (0/1/2/3)	NACT cycles (≤4/>4)
						0	I	II	III		
Yuan et al. (26)	TG: 64 [57.8, 68.3] PG: 63 [58, 68]	138/160	TG: 22.9 [21.1, 25.3] PG: 23.1 [21.1, 25.9]	TG: 68/12 PG: 69/11	TG: 22/58 PG: 26/54	TG: 4 PG: 4	TG: 21 PG: 27	TG: 28 PG: 26	TG: 27 PG: 23	TG: 6/14/33/27 PG: 5/15/29/31	TG: 69/11 PG: 70/10
Lu et al. (27)	TG: 63 [59.5, 68.2] PG: 62 [58, 67]	196/224	TG: 24.1 [21.4, 25.9] PG: 23.4 [21.5, 26]	TG: 108/14 PG: 106/16	TG: 85/37 PG: 80/42	TG: 20 PG: 19	TG: 9 PG: 15	TG: 40 PG: 30	TG: 23 PG: 58	TG: 6/19/72/25 PG: 21/13/30/58	TG: 106/16 PG: 105/17
Gu et al. (25)	TG: 65 [61, 69] PG: 63 [58, 66]	94/110	TG: 23.3 [21.7, 25.8] PG: 24 [21, 25.5]	TG: 66/5 PG: 36/3	NA	NA	NA	NA	NA	TG: 5/10/37/19 PG: 5/5/22/7	NA
Sugoor et al. (15)	TG: 63 [58, 66] PG: 65 [61, 69]	61/75	NA	NA	NA	TG: 6 PG: 2	TG: 3 PG: 9	TG: 11 PG: 15	TG: 13 PG: 16	NA	NA
Chen et al. (28)	TG: 61 [55, 67]	122/150	NA	NA	NA	NA	NA	NA	NA	TG: 19/22/81/28	NA

ASA, American society of Aneshesiologists; BMI, body mass index; CCI, Charlson comorbidity index; NA, not available; NACT, Neoadjuvant chemotherapy treatment; PG, proximal gastrectomy; TG, total gastrectomy; TRG, tumor regression grade.

### Surgical and pathological outcomes

**Figure 4:**
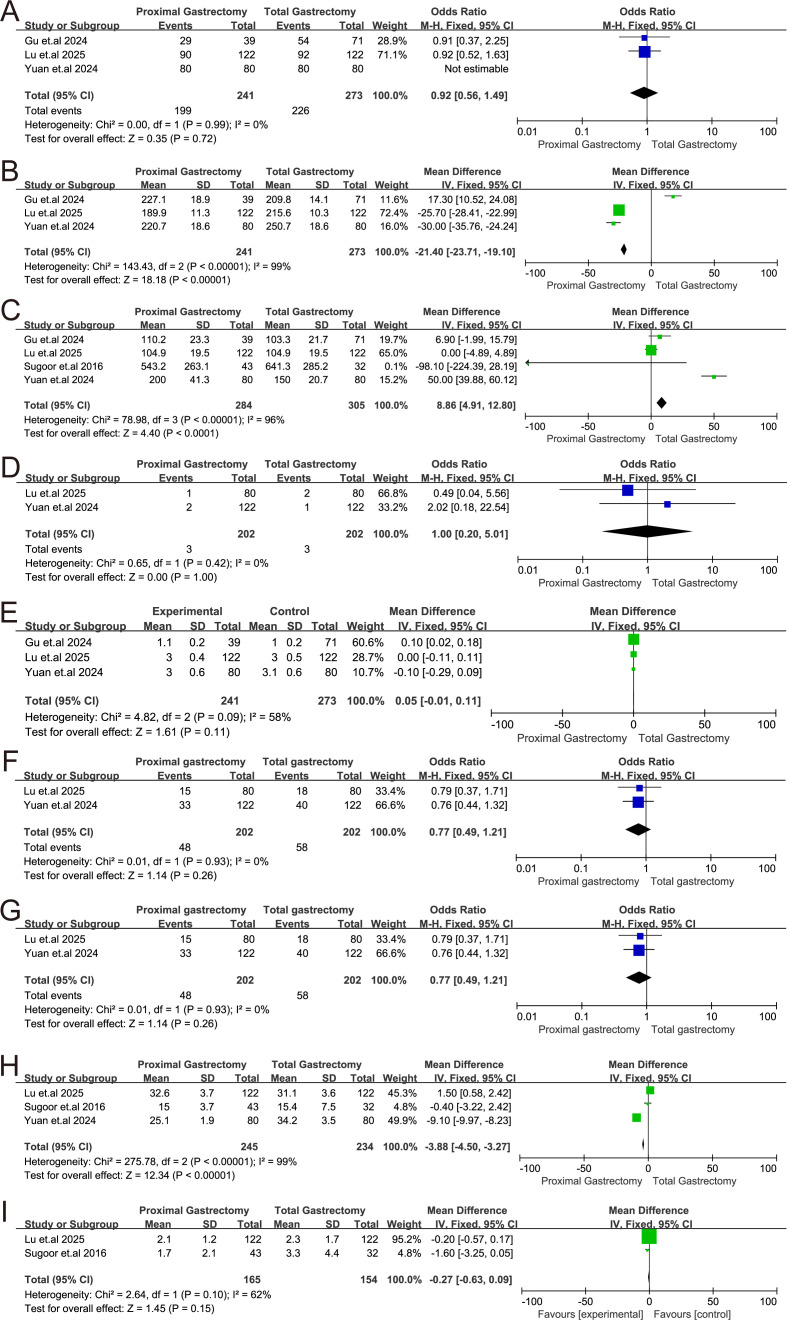
Forest plot of operative outcomes between PG and TG groups. **(A)** Adoption rate of minimally invasive techniques (laparoscopic or robotic-assisted). **(B)** Operative time. **(C)** Estimated blood loss. **(D)** Rate of positive resection margins. **(E)** Tumor diameter. **(F)** Perineural invasion rate. **(G)** Vascular invasion rate. **(H)** Total lymph node harvest. **(I)** Number of metastatic lymph nodes.

**Figure 5:**
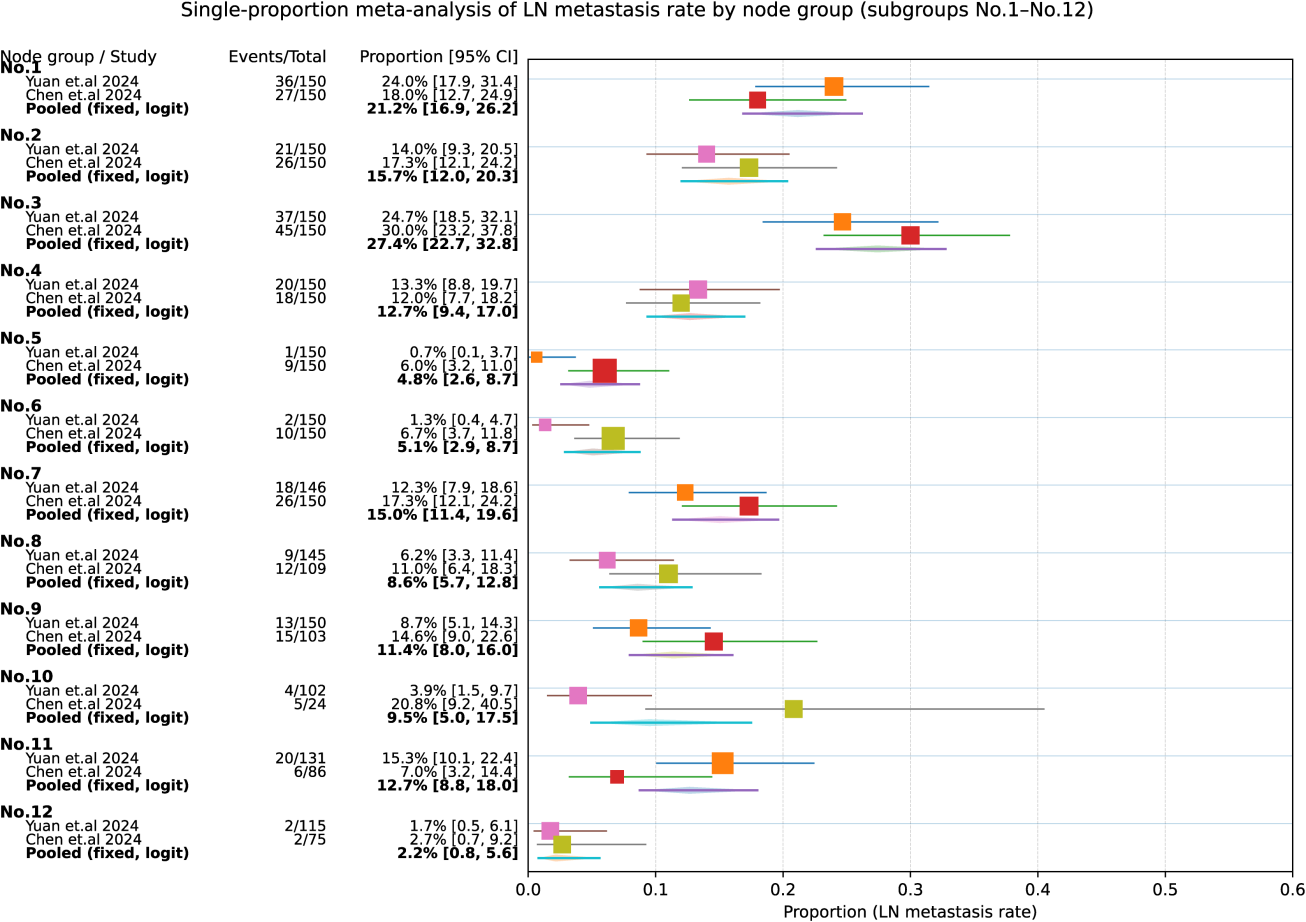
Forest plot of lymph node metastasis patterns in TG group.

Operative and pathological outcomes are summarized in **Table 3**. The use of minimally invasive techniques, including laparoscopic or robotic-assisted surgery, was comparable between the PG and TG groups (OR 0.92, 95% CI 0.56 to 1.49; I² = 0%, P = 0.99) (**Figure 4A**). PG was associated with a shorter operative time (MD −21.4 min, 95% CI −23.71 to −19.10; I² = 99%, P < 0.001) but slightly greater estimated blood loss (MD 8.86 mL, 95% CI 4.91 to 12.80; I² = 96%, P < 0.001) (**Figures 4B, C**). The positive resection margin rate did not differ significantly between groups (OR 1.00, 95% CI 0.20 to 5.01; I² = 0%, P = 0.42) (**Figure 4D**). Postoperative pathological findings were also broadly comparable. No significant differences were observed in tumor diameter (MD 0.05 cm, 95% CI −0.01 to 0.11; I² = 58%, P = 0.09), perineural invasion (OR 0.77, 95% CI 0.49 to 1.21; I² = 0%, P = 0.93), or vascular invasion (OR 1.00, 95% CI 0.20 to 5.01; I² = 0%, P = 0.93) (**Figures 4E–G**). Regarding lymph node dissection, PG was associated with a lower total lymph node harvest than TG (MD −3.88, 95% CI −4.50 to −3.27; I² = 99%, P < 0.001) (**Figure 4H**). However, the number of metastatic lymph nodes was similar between groups (MD −0.27, 95% CI −0.63 to 0.09; I² = 62%, P = 0.10) (**Figure 4I**). To further contextualize the oncological relevance of distal lymph node dissection, TG-cohort data were analyzed for distal lymph node metastasis patterns. The pooled metastasis rates were 12.7% (95% CI 9.4 to 17.0) for station #4, 4.8% (95% CI 2.6 to 8.7) for station #5, 5.1% (95% CI 2.9 to 8.7) for station #6, and 2.2% (95% CI 0.8 to 5.6) for station #12 (**Figure 5**). The complete lymph node metastasis patterns for the TG cohort are shown in **Table 4**.

**Table 3:** Surgical Characteristics of TG vs. PG for LAPGC after PSM.

Study	Operative time, min*	Blood loss, ml*	Tumor size*	Differentiation (Poor)	Dissected LNs*	Positive LNs*	Positive surgical margin*	Perineural invasion	Vascular invasion	Surgical approach	
										Minimally invasive	Open
Yuan et al. (26)	TG: 250 (210-300) PG: 220 (180-270)	TG: 150 (100-200) PG: 200 (100-300)	TG: 3 (2.1-5) PG: 3 (1.5-4.6)	TG: 43 (53.8) PG: 44 (55)	TG: 34.0 (27.0-44.0) PG: 25.0 (21.0-30.0)	NA	TG: 2 (2.5) PG: 1 (1.3)	TG: 14 (17.5) PG: 12 (15.0)	TG: 18 (22.5) PG: 15 (18.8)	TG: 14 (17.5) PG: 12 (15.0)	TG: 14 (17.5) PG: 12 (15.0)
Lu et al. (27)	TG: 215 (195.00-248.75) PG: 189.50 (169.25-227.25)	TG: 100.00 (100.00-200.00) PG: 100.00 (100.00-200.00)	TG: 3 (2-4.5) PG: 3 (2-4)	TG: 72 (59) PG: 61 (50)	TG: 34.0 (27.0-44.0) PG: 32.50 (23.75-43.00)	NA	TG: 1 (0.82) PG: 2 (1.64)	TG: 45 (36.9) PG: 39 (32.0)	TG: 40 (32.8) PG: 33 (27.0)	TG: 45 (36.9) PG: 39 (32.0)	TG: 45 (36.9) PG: 39 (32.0)
Gu et al. (25)	TG: 208 (187-254) PG: 225 (195-276)	TG: 100 (72-175) PG: 100 (100-200)	TG: 1 (1-2) PG: 1 (1-2)	TG: 47 (66.2) PG: 19 (48.7)	NA	TG: 2 (0-8) PG: 2 (0-5.25)	NA	TG: 24 (33.8) PG: 9 (23.1)	NA	TG: 24 (33.8) PG: 9 (23.1)	TG: 24 (33.8) PG: 9 (23.1)
Sugoor et al. (15)	NA	TG: 600 (200-1300) PG: 500 (150-1300)	NA	TG: 21 (65.6) PG: 16 (37.2)	TG: 15 (2-31) PG: 14 (1-27)	TG: 1 (1-18) PG: 1 (0-9)	NA	NA	NA	NA	NA
Chen et al. (28)	NA	NA	TG: 5.8 (4.6-8)	TG: 102 (68)	NA	NA	NA	NA	NA	NA	NA

*represents the median (interquartile range). LN, Lymph nodes; NA, not available; PG, proximal gastrectomy; TG, total gastrectomy.

**Table 4:** Incidence of lymph node metastasis in patients received total gastrectomy.

Study	Incidence of lymph node metastasis											
	#1	#2	#3	#4	#5	#6	#7	#8	#9	#10	#11	#12
Yuan et al. (26)	24% (36/150)	14% (21/150)	24.7% (37/150)	13.3% (20/150)	0.67% (1/150)	1.33% (2/150)	12.33% (18/146)	6.21% (9/145)	8.67% (13/150)	3.92% (4/102)	15.2% (20/131)	1.74% (2/115)
Chen et al. (28)	18% (27/150)	17.3% (26/150)	30% (45/150)	12% (18/150)	6% (9/150)	6.7% (10/150)	17.3% (26/150)	11% (12/109)	14.6% (15/103)	20.8% (5/24)	7% (6/86)	2.7% (2/75)

### Postoperative morbidity and recovery

**Figure 6:**
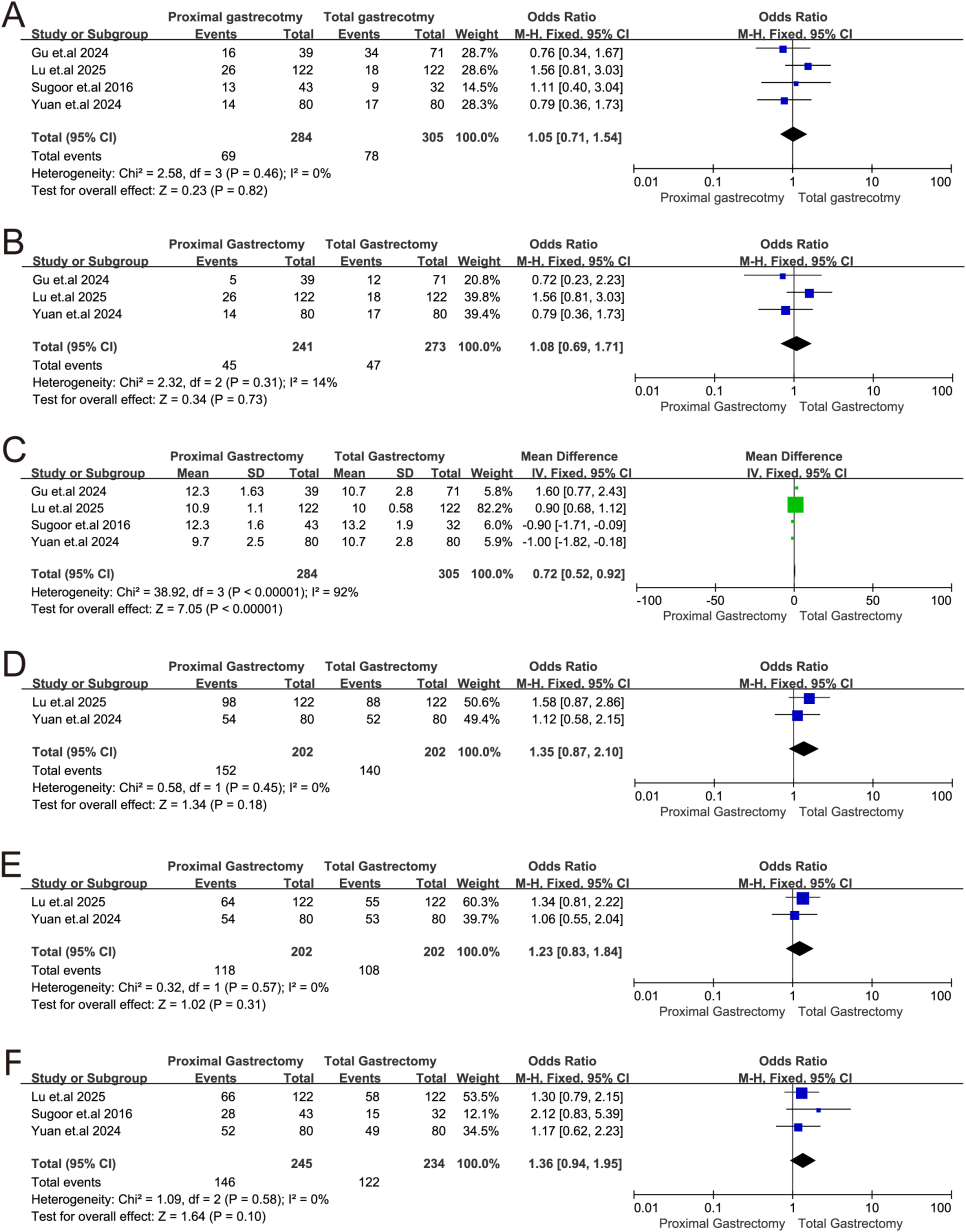
Forest plot of postoperative morbidity, recovery, and long-term oncological outcomes between PG and TG groups. **(A)** Overall incidence of postoperative complications. **(B)** Rate of Clavien-Dindo grade ≥ II complications. **(C)** Length of hospital stay. **(D)** Rate of adjuvant chemotherapy administration. **(E)** 5-year overall survival. **(F)** 5-year recurrence-free survival.

Postoperative morbidity and recovery outcomes are summarized in **Figure 6**; **Table 5**. The overall incidence of postoperative complications did not differ significantly between PG and TG (OR 1.05, 95% CI 0.71 to 1.54; I² = 0%, P = 0.46) (**Figure 6A**). Similarly, no significant difference was observed in Clavien-Dindo grade ≥II complications (OR 1.08, 95% CI 0.69 to 1.71; I² = 0%, P = 0.165) (**Figure 6B**). Length of hospital stay was longer in the PG group than in the TG group (MD 0.72 days, 95% CI 0.52 to 0.92; I² = 92%, P < 0.001) (**Figure 6C**). The rate of adjuvant chemotherapy administration was comparable between the two groups (OR 1.35, 95% CI 0.87 to 2.10; I² = 0%, P = 0.45) (**Figure 6D**), suggesting similar postoperative feasibility of subsequent systemic therapy.

**Table 5:** Short- and long-term outcomes of TG vs. PG after PSM.

Study	Early complications, n (%)				Hospital stays, d *	Adjuvant chemotherapy, n (%)	5-year OS	5-year RFS
	CD grade≥II	CD grade II	CD grade III	CD grade IV				
Yuan et al. (26)	TG: 17 (21.3) PG: 14 (17.5)	NA	NA	NA	TG: 10 (8-14) PG: 10 (8-12)	TG: 52 (65.0) PG: 54 (67.5)	TG: 68.40% PG: 68.40%	TG: 61.90% PG: 64.80%
Lu et al. (27)	TG: 18 (14.8) PG: 26 (21.3)	TG: 9 (7.38) PG: 15 (12.30)	TG: 8 (6.56) PG: 10 (8.20)	TG: 1 (0.82) PG: 1 (0.82)	TG: 10 (9-12) PG: 11 (8-13.8)	TG: 88 (72.1) PG: 98 (80.3)	TG: 45.50% PG: 52.70%	TG: 47.60% PG: 54.30%
Gu et al. (25)	TG: 34 (47.9) PG: 16 (41.0)	TG: 20 (28.2) PG: 9 (23.1)	TG: 11 (15.5) PG: 3 (7.7)	TG: 1 (1.4) PG: 3 (7.7)	TG: 13 (10-19) PG: 12 (10-17)	NA	P = 0.47	NA
Sugoor et al. (15)	TG: 9 (28.1) PG: 13 (30.2)	TG: 1 (3.1) PG: 2 (4.6)	TG: 3 (9.3) PG: 4 (9.2)	TG: 0 (0) PG: 0 (0)	TG: 10 (7-18) PG: 9 (7-18)	NA	NA	NA

* represents the median (interquartile range). CD, Clavien-Dindo; NA, not available; PG, proximal gastrectomy; PSM, propensity score-matched; OS, overall survival; RFS, recurrence-free survival; TG, total gastrectomy.

### Long-term oncological outcomes

Pooled analysis showed no significant difference in 5-year OS between PG and TG (HR 1.23, 95% CI 0.83 to 1.84; I² = 0%, P = 0.57) (**Figure 6E****;** **Table 5**). Similarly, 5-year RFS did not differ significantly between the two groups (HR 1.36, 95% CI 0.94 to 1.95; I² = 0%, P = 0.58) (**Figure 6F****;** **Table 5**). These findings suggest that PG may achieve long-term oncological outcomes comparable to those of TG in selected patients with locally advanced proximal gastric cancer following NAC.

### Sensitivity analyses

**Figure 7:**
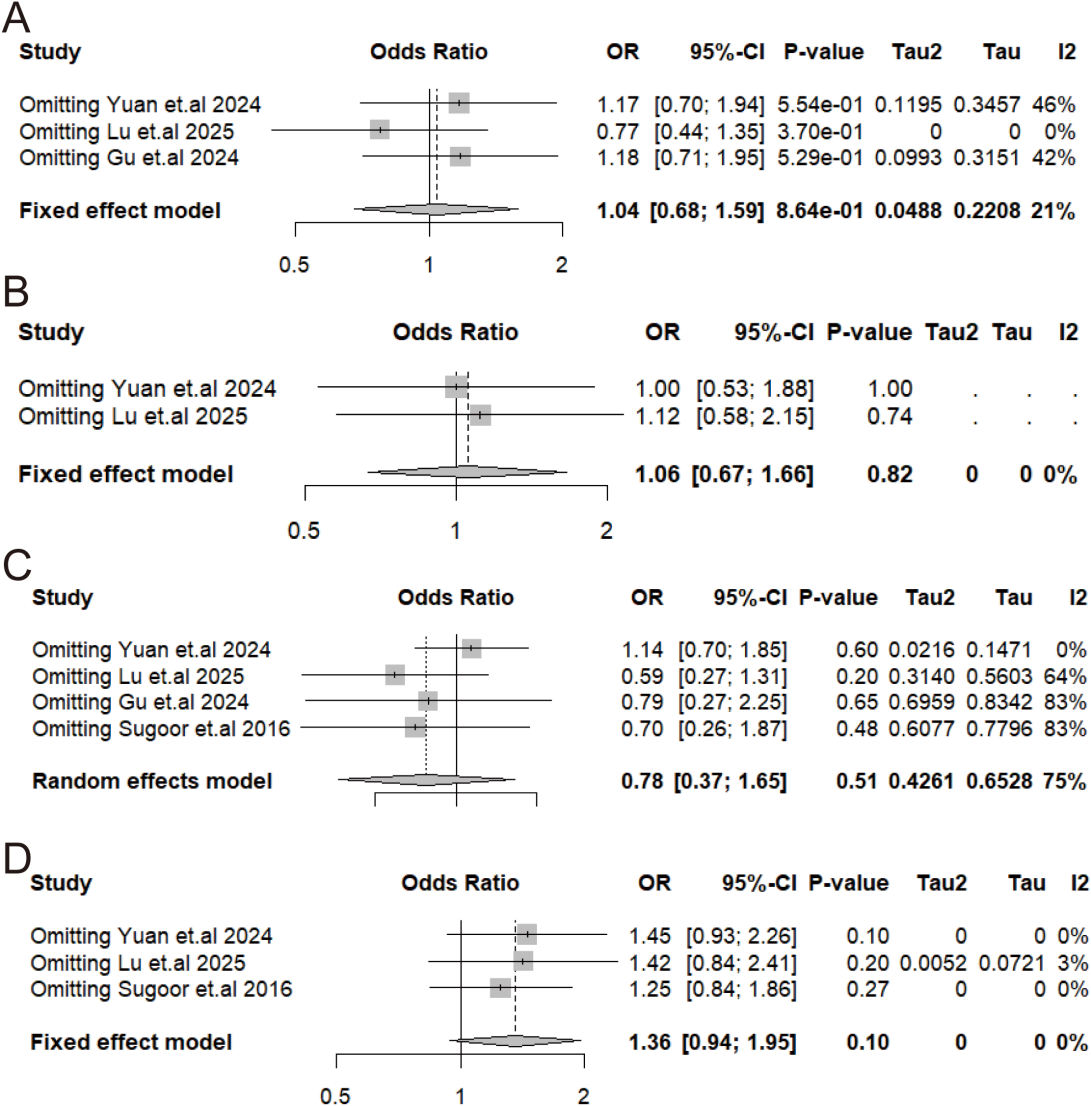
Sensitivity analysis for **(A)** overall postoperative complications. **(B)** Clavien-Dindo grade ≥ II complications. **(C)** 5-year overall survival. **(D)** 5-year recurrence-free survival.

Sensitivity analyses were performed by sequentially excluding individual studies to assess the stability of the pooled estimates. The effect estimates for OS (**Figure 7A**), RFS (**Figure 7B**), and overall postoperative complications (**Figure 7C**) remained directionally consistent and statistically non-significant regardless of which study was omitted. However, the pooled estimate for Clavien-Dindo grade ≥II complications showed some variability after study omission (**Figure 7D**), suggesting that this outcome should be interpreted with caution. Overall, the sensitivity analyses supported the stability of the main survival findings and the overall consistency of the comparative results.

## Discussion

The optimal extent of gastrectomy for PAGC following NAC remains controversial, largely due to the need to balance oncological radicality with postoperative function and quality of life. In this systematic review and meta-analysis of PSM studies, PG showed long-term oncological outcomes comparable to those of TG, with similar postoperative morbidity profiles. No significant differences were observed in 5-year OS or RFS, nor in overall or clinically significant postoperative complications, despite the less extensive nature of proximal resection. Collectively, these findings provide clinically relevant evidence supporting the potential oncological feasibility and perioperative safety of PG in selected patients with PAGC after NAC.

The comparable long-term survival between PG and TG represents the most consequential observation of this analysis. TG has traditionally been favored for PAGC because of concerns regarding inadequate resection margins and insufficient lymph node clearance after limited resection (7, 15, 25, 26). However, our findings suggest that, in the era of neoadjuvant treatment, long-term outcomes may not be determined solely by the extent of gastric resection. NAC can downstage the primary tumor, reduce nodal tumor burden, and eradicate micrometastatic disease, thereby potentially attenuating the theoretical oncological advantage of a more extensive surgical approach (13, 27, 28). In addition, the comparable rates of adjuvant chemotherapy administration between PG and TG further support the clinical feasibility of PG. Preservation of postoperative physiological reserve may be important for timely initiation and completion of systemic therapy, which remains a key component of treatment for advanced disease (7, 16, 32, 33). In this context, PG may provide adequate disease control without compromising long-term outcomes in appropriately selected patients.

Lymph node dissection remains a central concern when considering PG for PAGC, mainly because of the perceived risk of insufficient lymphadenectomy and compromised oncological radicality (18, 28, 29, 34, 35). In the present analysis, PG was associated with a lower total lymph node yield than TG. However, the number of metastatic lymph nodes did not differ significantly between the two procedures, suggesting that the reduced nodal harvest in PG may reflect anatomical and procedural differences rather than oncological inadequacy or systematic understaging. Importantly, in the context of NAC, both our pooled analysis and available NAC-specific studies showed a low incidence of metastasis in distal lymph node stations routinely dissected during TG but generally omitted or incompletely dissected during PG, particularly stations #4d, #5, #6, and #12a (26, 28). These findings suggest that distal nodal involvement remains uncommon in PAGC after NAC and may contribute limited therapeutic benefit in selected patients.

Several large retrospective studies that did not explicitly report NAC status provide complementary anatomical and biological support for this observation. Yura et al. (35) documented exceptionally low metastasis rates in stations #4d, #5, #6, and #12a among patients with T2–T3 proximal gastric cancer, while Sasako et al. (36) reported the lowest therapeutic indices at stations #5 and #6 for upper-third tumors compared with more distal lesions. Similar patterns of minimal distal nodal involvement were also observed in T3 proximal tumors by Ooki et al. (37) and Haruta et al. (34). Anatomically, lymphatic spread from proximal gastric tumors predominantly follows the left gastric artery pathway, involving stations #1, #3, and #7, whereas the right gastric and right gastroepiploic pathways represent more distant regional drainage routes that are less frequently involved during early or intermediate metastatic dissemination. This anatomical pattern may persist after systemic therapy and could explain the low metastatic rates observed at distal stations following NAC. Collectively, these findings support a more selective approach to lymphadenectomy in carefully selected patients, particularly when the expected therapeutic yield of distal node dissection is low. A more selective, biology-driven approach to lymphadenectomy may therefore be justified, particularly when weighed against the increased morbidity associated with more extensive resection. Furthermore, emerging predictive tools, including deep learning–based radiomics nomograms for estimating tumor regression and response to NAC, may further refine individualized surgical planning and help optimize the extent of lymph node dissection in PAGC (38, 39).

Postoperative complication profiles were broadly comparable between PG and TG, with no significant differences in overall or clinically significant complications. These findings suggest that, despite the technical complexity of reconstruction, PG does not appear to increase perioperative risk in selected patients. A modestly longer hospital stay was observed in the PG group; however, this difference is unlikely to reflect major surgery-related morbidity, given the comparable complication rates. Instead, it may reflect more cautious postoperative management during functional adaptation, concerns regarding reflux-related symptoms or delayed gastric emptying, variability in reconstructive techniques, and differences in institutional perioperative care pathways. Importantly, the longer length of stay did not translate into higher morbidity or reduced feasibility of subsequent oncological treatment.

Beyond early perioperative outcomes, functional recovery and nutritional preservation are important considerations when selecting the extent of gastrectomy. Function-preserving surgery may confer long-term benefits that are not fully captured by conventional morbidity endpoints. Evidence from randomized and retrospective studies has suggested that PG may be associated with improved postoperative nutritional recovery compared with TG, including better preservation of body weight, serum albumin levels, and micronutrient status (7, 29, 40). Patient-reported outcome analyses have also shown favorable quality-of-life profiles after PG, particularly in eating-related function, physical well-being, and global health status (41–44). Preservation of partial gastric reservoir function and digestive continuity may facilitate stabilization of oral intake and attenuate post-gastrectomy syndromes, thereby contributing to improved long-term functional outcomes (20, 45).

Several limitations should be acknowledged. First, the number of eligible studies was limited, which restricted the ability to conduct detailed subgroup analyses according to tumor stage, response to NAC, reconstruction method, or lymph node dissection strategy. Second, all included comparative studies were retrospective. Although PSM was used to balance measured baseline characteristics, residual confounding from unmeasured variables cannot be fully excluded. Third, publication bias could not be reliably assessed because only four comparative studies were included; therefore, funnel plots and formal asymmetry assessments were not considered informative and were not used to draw conclusions regarding publication bias. Future prospective studies and randomized trials are warranted to validate these findings and to establish standardized criteria for selecting candidates for PG after NAC, with particular emphasis on pathological response, nodal regression patterns, and long-term functional and nutritional outcomes.

## Conclusion

For PAGC treated with NAC, PG achieves oncological outcomes comparable to TG without increasing perioperative morbidity. The low incidence of distal lymph node metastasis observed in TG-cohort analyses provides contextual support for the oncological feasibility of PG in appropriately selected patients. By preserving gastric function, PG may offer potential advantages in postoperative recovery and long-term nutritional and functional outcomes. Further prospective studies are warranted to validate these findings and refine selection criteria for PG after NAC.

## Data Availability

The original contributions presented in the study are included in the article/**Supplementary Material**. Further inquiries can be directed to the corresponding author/s.
