# Supplementary material for: Proximal vs. total gastrectomy for proximal advanced gastric cancer after neoadjuvant chemotherapy: a systematic review and meta-analysis of propensity score-matched studies
Source: Front Oncol. 2026 Jun 2;16:1805000. doi: 10.3389/fonc.2026.1805000 (PMC13268900; doi:10.3389/fonc.2026.1805000)
Supplement: Supplementary file 1 [file Table1.docx]

**Table S1. Detailed Search Strategies**

| **Database** | **Search Date** | **search strategy** |
| --- | --- | --- |
| PubMed | From inception to December 31, 2025 | ("Stomach Neoplasms"[Mesh] OR "Gastric Cancer"[tiab] OR "Gastric Carcinoma"[tiab] OR "Cancer of Stomach"[tiab])  AND  ("Gastrectomy"[Mesh] OR "Proximal Gastrectomy"[tiab] OR "Total Gastrectomy"[tiab] OR "TG"[tiab] OR "PG"[tiab])  AND  ("Neoadjuvant Therapy"[Mesh] OR "Antineoplastic Agents"[Mesh] OR "neoadjuvant chemotherapy"[tiab] OR "NAC"[tiab] OR "preoperative chemotherapy"[tiab] OR "perioperative chemotherapy"[tiab]) |
| Embase | 1974 to December 31, 2025 | 1. exp stomach tumor/ or (gastric cancer or gastric carcinoma or stomach neoplas*).tw.  2. exp gastrectomy/ or (proximal gastrectomy or total gastrectomy or pg or tg).tw.  3. exp neoadjuvant therapy/ or exp antineoplastic agent/ or (neoadjuvant chemotherap* or nac or preoperative chemotherap* or perioperative chemotherap*).tw.  4. 1 and 2 and 3 |
| Cochrane Library | All years, searched on December 31, 2025 | #1 MeSH descriptor: [Stomach Neoplasms] explode all trees  #2 (gastric cancer or gastric carcinoma):ti,ab,kw  #3 #1 or #2  #4 MeSH descriptor: [Gastrectomy] explode all trees  #5 (proximal gastrectomy or total gastrectomy or PG or TG):ti,ab,kw  #6 #4 or #5  #7 MeSH descriptor: [Antineoplastic Agents] explode all trees  #8 (neoadjuvant chemotherap* or NAC or preoperative chemotherap*):ti,ab,kw  #9 #7 or #8  #10 #3 and #6 and #9 |
| Web of Science | All years, searched on December 31, 2025 | TS=("gastric cancer" OR "gastric carcinoma" OR "stomach neoplas*")  AND  TS=("proximal gastrectomy" OR "total gastrectomy" OR PG OR TG)  AND  TS=("neoadjuvant chemotherap*" OR "NAC" OR "preoperative chemotherap*" OR "perioperative chemotherap*") |
